# Pyoderma Gangrenosum in Two Successive Pregnancies Complicating Caesarean Wound

**DOI:** 10.1155/2014/654843

**Published:** 2014-02-11

**Authors:** Sapna Vinit Amin, Neha Bajapai, Ashwini Pai, Sunanda Bharatnur, Shripad Hebbar

**Affiliations:** Department of Obstetrics and Gynaecology, Kasturba Medical College, Manipal University, Manipal 576 104, India

## Abstract

Pyoderma gangrenosum (PG) is a rare ulcerative cutaneous disorder with tendency to recur in the injured area. Though most of the time is associated with chronic systemic conditions, it can occur in isolation and can be a diagnostic dilemma. The aetiology is poorly understood. The diagnosis is based on clinical features and excluding other causes of skin ulcers, as it does not have characteristic histopathology or laboratory findings. Lesions can develop after surgery, after trauma or de novo. We are reporting a 32-year-old pregnant lady with two previous instances of pyoderma gangrenosum in the previous pregnancy, who in postoperative period following caesarean section developed the same condition for the third time. She responded well to local wound care, oral Prednisolone, and Dapsone and made a good recovery. Pregnancy being an immunologically altered status can play a role in development of pyoderma gangrenosum and one should always rule out its possibility when there is a delayed wound healing.

## 1. Introduction

Brunsting et al. in 1930 described clinical features of a rare cutaneous ulcerative condition in 5 cases occurring in adults [1]. They initially thought that lesions were produced by *Streptococcus *species and named the condition pyoderma gangrenosum, a purulent infection of cutis produced by pyogenic bacterial species. Nevertheless, its aetiology is poorly understood. Though there are good numbers of cases reported from other surgical sites, its occurrence in pregnancy is sparingly published [2]. The condition is associated with systemic diseases in at least 50% of patients who are affected [3]. Pregnancy being a state of immunosuppression may be affected in susceptible individuals. The diagnosis is made by excluding other causes of similar appearing cutaneous ulcerations, including infection, malignancy, vasculitis, collagen vascular diseases, diabetes, and trauma [4]. 30% of affected individuals give history of prior trauma or injury to the ski. The prognosis of pyoderma gangrenosum is generally good; however, recurrences may occur and residual scarring is common. Therapy of pyoderma gangrenosum involves the multidisciplinary approach and use of anti-inflammatory and immunosuppressive agents.

## 2. Case Report

**Figure 1:**
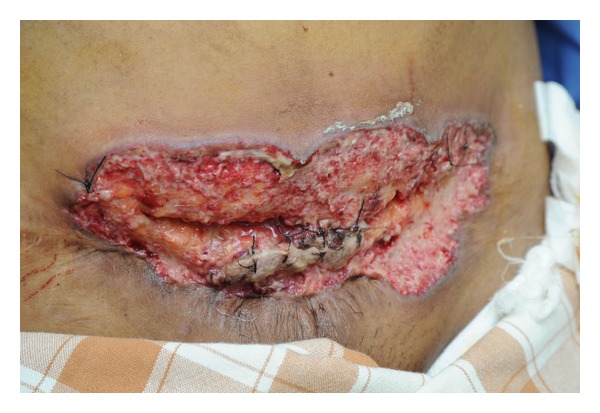
Appearance of caesarean wound on 9th post-op day.

A 32-year-old 2nd gravida with previous lower segment caesarean section (LSCS) and history of pyoderma gangrenosum during her first pregnancy presented to university hospital at 32 weeks of gestation with threatened preterm labour. She had supervised uncomplicated pregnancy elsewhere. Patient received steroid prophylaxis. She complained of fresh vaginal bleeding and obstetric ultrasound revealed abruptio placenta. In view of previous scar and as cervix was unfavourable, she was taken up for emergency caesarean delivery. A live male baby of 1900 g was delivered, with an APGAR score of 9. Placental examination revealed a retroplacental clot of 150 grams. Intraoperative period, otherwise, was uneventful and concurrent Pomeroy's tubectomy was done. Postoperatively patient had fever, tachycardia, and tachypnoea on 2nd day. From the 3rd postoperative day onwards, sloughing and purulent discharge were noted from the surgical wound. There were bilateral crepitations in both of the lung fields. Chest X-ray showed bilateral middle zone opacities. Subsequently she progressed to adult respiratory distress syndrome (ARDS) and required care in intensive care unit (ICU) for two days. Fever spikes continued and Inj. Ceftriaxone was changed to Injectable Piperacillin and Tazobactam combination 4.5 gm 8th hourly. Wound gaping of superficial layers was present (Figure 1). Wound swab culture was sterile. Hemoglobin and blood sugars of the patient were normal. There was mild polymorphonuclear leukocytosis. Dermatological opinion was sought and a diagnosis of recurrent pyoderma gangrenosum in pregnancy was made. As per their advice, patient received systemic steroids (Tab Prednisolone 20 mg two times daily) and Tab Dapsone 100 mg in divided doses and parenteral antibiotics were continued. Thrice daily dressing with saline, betadine, and metronidazole ointment was done. On the 31st postoperative day, complete wound healing occurred by secondary intention and at present she is doing fine.

In her first pregnancy which dated 9 years ago, she presented at 32 weeks of gestation with complaints of fever and a large ulcer in the right leg to the Department of Surgery in our hospital. Case record was obtained from Medical Record Department. Ulcer had developed spontaneously over a short duration of 10 days and there was no history of trauma. Examination revealed a 20 cm ulcer on anterior aspect of right leg with sloughing and exuberant pale granulation tissue and violaceous margins. Biopsy from the edge of ulcer was taken and was reported as pyoderma gangrenosum. Antinuclear antibody (ANA) profile was negative. In spite of intravenous antibiotics, she had persistent fever and developed nonassuring foetal heart tracings after two weeks. She was taken up emergency caesarean section in view of worsening foetal heart traces. On the 3rd postoperative day she developed extensive sloughing and necrosis of the caesarean wound, and she was started on T. Dapsone 100 mg/day in divided doses. She responded dramatically and healing of both leg ulcer and surgical wound was satisfactory. Patient was discharged on the 10th postoperative day and did not develop any recurrence until present episode.

## 3. Discussion 

Pyoderma gangrenosum is one of the rarest noninfectious neutrophilic dermatoses. Majority of cases belong to the age group of 20 to 50 years, with women being more often affected than men. Lesions can develop spontaneously, after surgery or after minor trauma [5]. The latter form is known as the pathergy phenomenon. Lesions can be single or multiple, chronic or recurrent. There can be severe wound pain, fever, and malaise. The classical presentation is the development of an erythematous papule or pustule that breaks down to form an ulcer with purulent discharge, as well as violaceous colored borders spreading peripherally and overhanging the ulcer bed. The early stages may resemble a bacterial infection. The diagnosis is made by exclusion of other cutaneous conditions. Histopathology of biopsy taken from ulcer edge shows a deep suppurative folliculitis with dense neutrophilic infiltrate [6]. Treatment includes topical therapies like local wound care and dressings, topical cromolyn sodium 2% solution, and 5-aminosalicylic acid [7]. Based on empirical findings, first choice of treatment is systemic high dose corticosteroids. A dramatic improvement with corticosteroid treatment supports the diagnosis of pyoderma gangrenosum. In mild cases topical therapy might be sufficient. Corticosteroids, like Prednisolone, 1 to 2 mg per kg per day, are widely used for initial therapy [7]. Immunosuppressive therapy with cyclosporin has become an accepted treatment for widespread PG after initial steroids or in combination with steroids [8].

Dapsone (diaminodiphenylsulfone), a drug used for leprosy, is another popular drug used in treatment of pyoderma gangrenosum in combination with steroids [7]. It is given in the doses of 100 to 200 mg per day in divided doses, with the only contraindication being abnormal glucose-6-phosphate dehydrogenase level. It is supposed to inhibit neutrophil migration and have some anti-inflammatory action. Those patients for whom steroids cannot be given for some reason can be given azathioprine (100 to 150 mg/day); however, a latency period of 2 to 4 weeks is normal before clinical response begins [9].

Recently infliximab in a single dose of 5 mg/kg given in day, thereafter repeated at 4 to 6 weeks intervals, has been shown to be effective. Intralesional cyclosporine, azathioprine, chlorambucil, mycophenolate, and several other drugs have been used with various success, but one has to exercise caution in breast feeding women [10].

When there is extensive tissue loss, autologous split skin graft can be tried, but it should be remembered that it can flare up the disease at the donor site. Use of bioengineered skin, like the dermal regeneration template Integra, hair follicle stem cell-derived autologous keratinocyte sheets Epidex, or hyaluronic acid-based autologous keratinocyte delivery system laser-skin is still experimental and not available in all the institutions [11].

## 4. Summary

We were fortunate enough as diagnosis and treatment were straight forward in this case after referring to previous inpatient medical record. However, when a postoperative ulcerative wound defect is not healing despite standard wound care, antibiotic treatment, and negative cultures, the possibility of pyoderma gangrenosum should be kept in mind. Early diagnosis and subsequent treatment are crucial for limiting scar tissue.
